# A global database of West Nile virus host prevalence and competence

**DOI:** 10.1038/s41597-026-07648-8

**Published:** 2026-06-26

**Authors:** Alex Richter-Boix, Júlia Froxán-Grabalosa, Nina Bogdanović, Catuxa Cerecedo-Iglesias, William Wint, Roya Olyzadeh, Tijmen Hartung, Giovanni Marini, Daniele Da Re, Marion P. G. Koopmans, Reina S. Sikkema, Frederic Bartumeus

**Affiliations:** 1 Centre d’Estudis Avançats de Blanes (CEAB-CSIC), Blanes, Spain; 2 Environmental Research Group Oxford Ltd, c/o Dept Biology, Oxford, United Kingdom; 3 Department of Viroscience and Pandemic & Disaster Research Center, Erasmus MC, Rotterdam, the Netherlands; 4 Research and Innovation Centre, Fondazione Edmund Mach, San Michele all’Adige (TN), Italy; 5 Institució Catalana de Recerca i Estudis Avançats (ICREA), Barcelona, Spain

## Abstract

West Nile virus (WNV) is one of the most widespread arboviruses globally and is maintained primarily through a bird-mosquito-bird transmission cycle, while other vertebrates play more limited roles. Host contributions to transmission depend on both infection evidence in natural populations (reflecting exposure and susceptibility) and reservoir competence, determined by the magnitude and duration of viraemia sufficient to infect mosquitoes. Despite extensive surveillance and experimental research, no comprehensive, standardised resource has integrated evidence on host exposure and infection in natural populations together with experimental data on host competence across vertebrate taxa. Here, we present two harmonised datasets compiled through a systematic literature review: (i) a WNV host prevalence dataset, summarising infection and serological evidence in wild and captive vertebrates; and (ii) a WNV host competence dataset, derived from controlled experimental infections. The prevalence dataset aggregates records from 541 studies across 91 countries (1950–2023), comprising 535,568 tested individuals from 1,801 vertebrate species. The WNV host competence dataset compiles 113 experimental infection studies covering 103 species and 3,030 individuals, and provides standardised time-resolved viraemia and survival data with accompanying metadata, enabling reconstruction and/or modelling of species-specific viraemia trajectories and the derivation of quantitative competence metrics. Both datasets use standardised taxonomy and incorporate synonym crosswalks to facilitate linkage with trait databases, phylogenetic trees and species distribution products. Together, these resources provide a unified foundation for macroecological analyses, surveillance gap assessment, and modelling multi-host WNV transmission dynamics.

## Background & Summary

The West Nile virus (WNV, Flaviviridae: Flavivirus) exemplifies the complex epidemiological and ecological dynamics associated with emerging global vector-borne diseases in the face of increasing environmental and anthropological change^1–3^. Originally endemic to Africa^4,5^ WNV has, over recent decades, undergone a remarkable expansion in both prevalence and geographic range. It is now established in Europe, Asia, the Americas and Oceania^6–10^. In particular, the expansion of WNV in the United States followed an initial introduction likely originating in the Middle East^11^. Its sustained spread across the Americas and globally has been largely driven by environmental and climate-related changes affecting vector and host distributions^12^.

As a multi-host, multi-vector pathogen, WNV has been isolated from more than 46 species of mosquitoes^13^, the genus *Culex* being the most representative^14^. The virus can infect a wide range of vertebrate hosts, including birds, mammals, reptiles, and amphibians, reflecting its high ecological flexibility and complexity^15–17^. WNV is maintained primarily through an enzootic transmission cycle involving *Culex* mosquitoes and avian reservoir hosts, with occasional epizootic and zoonotic spillover to a variety of dead-end hosts, most notably humans and equines, which can develop severe clinical disease.

The dynamics of WNV transmission is governed by the co-occurrence of competent mosquito vectors, susceptible vertebrate hosts, and the pathogen in climate and ecologically suitable environments^11,18–20^. In recent decades, empirical evidence has revealed substantial inter-specific variation in host exposure, susceptibility to infection, magnitude and duration of viraemia, and infection-associated mortality across a diverse range of vertebrate taxa^21–25^. Understanding host ranges and their transmission capacity for WNV is fundamental for identifying and understanding the ecological drivers of viral amplification, anticipating transmission dynamics, and developing proactive surveillance^26–29^.

Within the host range, the contribution of each species to WNV transmission is determined by three key factors: (1) its abundance; (2) its competence, or infectiousness, defined as the ability of a host to become infected and develop sufficient viraemia (both magnitude and duration) to subsequently infect feeding mosquitoes; and (3) its susceptibility and exposure, which are shaped by both the host’s immune response and its behavioral ecology, the latter influencing vector contact rates, which, in turn, are also modulated by mosquito feeding preferences^30–33^. Given the ability of WNV to infect a broad spectrum of vertebrate taxa that differ in prevalence (reflecting exposure and susceptibility) and in competence, assembling a comprehensive dataset of confirmed host species and their associated prevalence and competence is a crucial first step towards modeling transmission risk and improving preparedness and response strategies for WNV^22,34,35^. Such data sets can facilitate future investigations aimed at identifying key amplifying hosts, assessing gaps in host surveillance, and refining monitoring strategies. They can also contribute to improving our understanding of how ecological and evolutionary processes—such as the structure of the transmission network and host trait variability—shape the dynamics of WNV, ultimately supporting the development of One Health frameworks and strengthening early warning and prediction systems^36–38^.

Based on this rationale, the present study introduces the first unified global database that systematically compiles empirical data on vertebrate hosts involved in WNV transmission. Through a comprehensive systematic review, we compile an overview with information on both (1) host exposure and susceptibility, inferred from WNV prevalence data in wild populations, and; (2) host competence, based on experimental infection studies. Although previous studies addressed aspects of WNV host susceptibility within more limited taxonomic scopes^16,17,39^, no prior effort has consolidated these data into a unified and taxonomically broad database.

## Methods

### Study design and protocol

We conducted a systematic literature review to integrate and synthesize evidence related to WNV infection as a means of identifying potential hosts on a global scale. To achieve this, we developed two independent databases. The WNV host prevalence database compiles studies on the prevalence of WNV in non-human vertebrates, encompassing wild populations, domesticated animals, and those in zoological settings, where exposure and infection with WNV have been tested and confirmed. The WNV host competence database focuses on evaluating host competence by compiling experimental infection studies and extracting quantitative measures in a standardised format to enable reconstruction and/or modelling of species-specific viraemia curves. In both cases, we used a hybrid search strategy that combined database searches with snowballing, as this approach has proven to be more effective in identifying primary studies^40^.

### Literature search strategy and databases

To assess the prevalence of WNV in non-human vertebrate populations, this study builds on the quantitative review of avian field studies by Tolsá *et al*.^16^. Their database was critically reviewed and systematically expanded to include additional data on other vertebrate hosts, such as mammals, squamates, and amphibians, through an extensive search of the scientific literature. Literature searches were conducted in two phases, first in July 2023 and subsequently updated in March 2025, using the same search strategy and followed by systematic snowballing. The main academic databases consulted included PubMed (https://pubmed.ncbi.nlm.nih.gov/), ScienceDirect (https://www.sciencedirect.com/), Semantic Scholar (https://www.semanticscholar.org/), Google Scholar (https://scholar.google.com/), and SciELO (https://scielo.org/en/). Search strategies combined controlled vocabulary (where available) and free-text terms related to pathogen, host taxa, and study outcomes. The core search terms included “West Nile virus” OR “WNV”, combined with host-related terms (e.g. bird, avian, mammal, reptile, amphibian, host) and outcome-related terms reflecting prevalence, surveillance, and experimental infection (e.g. prevalence, seroprevalence, infection, surveillance, experimental, viremia, viral load, transmission, host competence). The literature search covered the period from the initial isolation of WNV in Uganda in 1937^4^ to 31/12/2024. The same academic databases were used to construct the WNV host competence database, focusing on data derived from experimental infection studies.

In both cases, articles retrieved from the database search were screened using predefined inclusion and exclusion criteria, as specified below. Subsequently, a snowball approach was employed to enhance the completeness of the data set. During backward snowballing, the reference lists of included articles were examined to identify earlier studies relevant to the review’s scope, with judgment based on article title and their contexts of citation. For forward snowballing, the titles of the included articles were searched in Google Scholar to locate citing publications. Citations were reviewed and full-text evaluations were performed based on the relevance indicated in the title and abstract. If the abstract did not provide sufficient information to determine whether a reference should be included or excluded, the full text was screened for eligibility. No restrictions were applied on publication type, year, region, or language to ensure inclusivity and minimize potential biases arising from study characteristics or linguistic barriers^41,42^. All potentially relevant articles were imported into Zotero (6.0.37)^43,44^ for screening and data extraction.

### WNV Host Prevalence Database

#### Data selection

The full text evaluation was selected according to the following inclusion criteria: (i) The publication contains data from epidemiological or prevalence studies of WNV in any infected animal in the wild, regardless of the type of diagnostic test used. We included studies reporting on the occurrence of WNV in wild animals (including those held in, e.g., wildlife rehabilitation centers), captive animals (e.g., zoological institutions) and domesticated animals from farms, breeding facilities or human-modified environments (e.g., dogs, cats, or pigeons); (ii) The study includes negative results for any diagnostic test for WNV in wild hosts to properly document which species and regions have been evaluated.

Publications were excluded if they met one or more of the following criteria: (i) The publication only indicates the species number of individuals that tested positive for WNV without reporting the total number of individuals tested (in case this information was mentioned but not provided; additional data were requested from the study authors to allow inclusion); (ii) The species were not identified at least at the genus level, with data presented only at the family or order level; (iii) Case reports of individual animals; (iv) Reports of suspected WNV infection based on clinical signs in individual animals without laboratory confirmation; (v) Wildlife outbreak or mass mortality reports attributed to WNV without laboratory confirmation of WNV in the event (e.g., no PCR/RT-PCR, virus isolation, or serological evidence); (vi) Publications focused on experimental infections; (vii) Studies involving human subjects; (viii) Studies for which the full text could not be obtained despite author contact attempts.

#### Data extraction

The data were extracted from selected articles into a pre-designed Microsoft Excel worksheet. The extracted data included: (i) Host species (scientific name), number of individuals sampled, number of animals tested positive, diagnostic method or methods applied, prevalence, lineage and strain of WNV (if reported), study area, sampling year and period, study area (country, province/region, zoorealms) and longitude and latitude. Because exact coordinates were rarely reported—most studies referenced a general locality—we assigned coordinates to the centroid of the municipality, province/region, or other area specified in the study. These coordinates were then used to assign observations to terrestrial zoogeographic realms, following distributions for birds, mammals, and other terrestrial vertebrates (21,037 species) delineated by Holt *et al*.^45^. (ii) Publication details (author name, year of publication, title, DOI). Whenever prevalence data were expressed as percentage, raw numbers were obtained, converting the percentages to the nearest integers.

To address taxonomic inconsistencies, the common names and species reported in the studies were standardized to align with current taxonomic conventions. For birds, we utilized four taxonomic frameworks to facilitate future integrative analyzes: BirdLife International^46^, BirdTree^47^, eBird^48^, and the GBIF Backbone Taxonomy with associated taxon IDs^49^. Taxon crosswalks established by Tobias *et al*.^50^ were used to harmonize the taxonomic frameworks. When necessary, we consult Avibase (The World Bird Database)^51^ and AviList (The Global Avian Checklist)^52^ for synonymies and name updates. For mammals, scientific names were verified against the GBIF Backbone Taxonomy^49^ and the Mammal Diversity Database^53^, while for reptiles and amphibians, we used the GBIF Backbone Taxonomy to ensure accuracy and consistency, retrieving both accepted scientific names and the corresponding GBIF taxon IDs. We included the species name, genus, specific epithet, family, order, class, phylum, and kingdom for each of the recorded taxa to ensure taxonomic resolution and facilitate downstream ecological analyzes.

Diagnostic methods for WNV detection were classified according to their methodological principles and diagnostic utility. In the prevalence dataset, each diagnostic record is represented in a separate row and linked to the same observation through a unique *observation_code*. To make diagnostic heterogeneity explicit and easier to interpret, we added five variables to each record: *diagnostic_method*, *assay_stage*, *workflow_design*, *diagnostic_group*, and *biological_interpretation*.

*diagnostic_method* identifies the specific assay reported in the source study. *assay_stage* describes the role of the assay within the diagnostic workflow, distinguishing assays used for (i) screening, (ii) confirmatory testing, or (iii) as independent assays not explicitly linked to a screening-confirmation sequence. *workflow_design* denotes the overall configuration of methods reported for observation, distinguishing records based on a single diagnostic method from those using sequential screening-confirmatory workflows or assays applied independently in parallel (e.g, a molecular test for active infection and a serological approach testing past exposure to the same samples). Observations containing both sequential and independent parallel components were classified as *workflow_design* = parallel. *diagnostic_group* classifies the test in more general terms as (i) serology, (ii) molecular, (iii) antigen, or (iv) isolation. *biological_interpretation* indicates whether a positive result is best interpreted as a past exposure, a recent exposure, or an active infection.

This structure makes explicit the implications of diagnostic heterogeneity for downstream analyses. In particular, serological assays mostly reflect exposure history, whereas molecular, antigen-based, and virus-isolation methods are more indicative of current or very recent infection. Therefore, raw prevalence estimates should not be directly compared between diagnostic categories without taking these differences into account.

**Molecular tests** include reverse transcription polymerase chain reaction (RT-PCR) and quantitative RT-PCR (qRT-PCR), which are widely used to detect WNV RNA in blood or cerebrospinal fluid^54,55^. These methods can offer high sensitivity and specificity for detecting viral RNA during the acute phase of infection, particularly in blood and cerebrospinal fluid, although sensitivity varies depending on the primers used (e.g., lineage-specific vs. pan-lineage). Their sensitivity declines as the disease progresses^56^, but alternative samples such as urine, swabs, brain tissue, or feathers may allow detection beyond the typical viraemic window^57^. In the dataset, these assays are recorded as diagnostic_method = molecular_detection, grouped as *diagnostic_group* = molecular, and interpreted as active infection. Their *assay_stage* is independent; depending on the observation, *workflow_design* may be single_method or parallel.

**Antigen-based tests** detect viral proteins in tissues or fluids, providing evidence of active viral replication. These include Immunohistochemical Methods (IHC) and field-deployable tests such as VecTest that offer more immediate indications of infection^55^. In the dataset, these assays are classified as *diagnostic_group* = antigen and are interpreted as active infection. They are usually annotated as independent and *workflow_design* may be single_method or parallel.

**Serological or antibody-based tests** detect IgM and IgG antibodies against WNV; IgM typically appear 3 to 7 days after exposure, while IgG antibodies appear shortly after. Among these, blocking ELISA tests are the most commonly used, with numerous commercial kits available^58,59^. ELISA tests are fast, but can exhibit cross-reactivity with antibodies against other flaviviruses -depending on the antigen used- complicating the interpretation of the results^60^. In the dataset, these assays are recorded as *diagnostic_method* = serological.ELISA, grouped as *diagnostic_group* = serology, and interpreted as past exposure. Depending on the study design, they are annotated as *assay_stage* = screening in sequential workflows or as *assay_stage* = independent when used alone or in parallel; accordingly, *workflow_design* may be single_method, sequential, or parallel.

Virus neutralisation tests (VNTs), including plaque reduction neutralisation tests (PRNT) and microneutralisation assays (micro-VNT), are slower and more technically demanding than ELISA, and typically require higher biosafety laboratory conditions; however, they are often regarded the gold standard for serological confirmation. Although not entirely free of cross-reactivity, they are considerably more specific than ELISA in distinguishing WNV from other flavivirus infections, making them an essential confirmatory tool for ELISA-positive cases^61–63^. These assays are recorded as *diagnostic_method* = serological.VNT, grouped as *diagnostic_group* = serology, and interpreted as past exposure. Their *assay_stage* may be confirmatory in sequential workflows, or independent when used alone or in parallel with a molecular method; accordingly, *workflow_design* may be single_method, sequential, or parallel.

Hemagglutination Inhibition (HI), an antibody-based method, detects antiviral antibodies by measuring the virus’s ability to inhibit red blood cell agglutination. Although HI is generally more specific than ELISA, particularly in distinguishing closely related flaviviruses, some cross-reactivity may still occur. The Microsphere Immunoassay (MIA) and other multiplexed ELISA-like diagnostic techniques use multiple antigens from different viruses to detect IgM and IgG antibodies in serum samples that offer high performance and scalability^64^. Multiplexed IgM and IgG MIA platforms demonstrate some error relative to neutralization assays, but offer high-throughput capabilities^63^. In the dataset, HI and MIA are classified within *diagnostic_group* = serology and interpreted as past exposure. HI may occur as an *assay_stage* = screening in sequential workflows or as an *assay_stage* = independent when used alone or in parallel, whereas MIA is currently annotated as independent.

IgM-specific serological assays are treated separately in the dataset because they are more informative about recent exposure than general antibody assays. These records are classified within serological methods but assigned as recent exposure under the *biological_interpretation* variable instead of past exposure. In the current dataset, IgM assays are annotated as *assay_stage* = independent; *workflow_design* may be single_method or parallel.

**Virus isolation methods** are also retained when reported. These provide direct evidence of infectious virus in the sample and are therefore classified as *diagnostic_group* = isolation with biological_interpretation = active_infection. Their *assay_stage* is *independent*; depending on the observation, *workflow_design* may be *single_method* or *parallel*.

### WNV Host Competence Database

#### Data selection

All the papers collected in the first steps using a combination of database search and snowballing techniques were exposed to a full text scan before including them in the review. Studies were required to meet specific criteria to be included: (i) Publications had to report data from controlled experimental studies investigating WNV infection in animals, providing detailed descriptions of experimental protocols, such as dose, infection method, and WNV lineage or strain. (ii) Studies were also required to specify the host species and the number of individuals infected. (iii) Quantitative laboratory results, mainly time-series viremia levels and mortality rates, should be clearly presented in the text, as tables, or in a graphical format. (iv) Additionally, studies needed to report the exposure status of individuals prior to infection, ensuring that free-living animals had not previously been infected with WNV or related virus, vaccinated or affected by other diseases, and that captive animals were not vaccinated. Studies investigating WNV vaccines were also included, as they often feature control groups of intentionally exposed unvaccinated individuals to the virus, offering comparative insights into immune responses and infection dynamics.

Studies were excluded if (i) they did not report key experimental results, such as viraemia levels or mortality rates, (ii) did not provide sufficient methodological detail, (iii) did not clearly identify host species, (iv) included vaccinated or previously infected animals, or (v) if the full text of the study could not be accessed.

#### Data extraction

Data were extracted from the included articles using a standardised extraction sheet created in Microsoft Excel. Two reviewers independently assessed the articles based on predefined selection criteria to ensure reliability and accuracy. The extracted information included: (i) publication details (title, first author, year and DOI); and (ii) results of the study details, such as host species and taxonomy (standardised by following the same procedure as for the WNV Host Prevalence Database), WNV lineage and strain used for infection, year of isolation and geographic origin of the WNV strain, GenBank accession number(s) for the corresponding viral sequence when available, virus dose and its corresponding units, sample size of infected individuals, viraemia dynamics expressed as log_10_ titres over time, and mortality data, recorded as the number of surviving individuals during the study period.

In the competence database, each row corresponds to a specific day post-inoculation within an experimental infection curve. Accordingly, the variable day refers to the specific day represented by that row, *sample_size_original* refers to the number of individuals inoculated at the start of the experiment, and *sample_size_survival* refers to the number of individuals still alive on that same row-specific day.

Daily viraemia levels, expressed as log_10_ titres, and mortality data were extracted from tables, figures, or supplementary materials from the included studies. Graphical data were digitised using WebPlotDigitizer^65^ by the same two extractors involved in the broader data extraction process.

To quantify uncertainty associated with manual digitisation, a validation subset of 25 viraemia curves was assessed in two ways. First, repeated extraction of the same curves by the same observer(s) was used to estimate within-observer digitisation error, yielding a mean absolute error of 0.032 log_10_ PFU (RMSE = 0.049 log_10_ PFU), with a maximum absolute deviation of 0.113 log_10_ PFU. Second, independent digitisation of the same subset by the two extractors was compared to assess between-observer agreement, producing a mean absolute error of 0.029 log_10_ PFU and a maximum absolute deviation of 0.242 log_10_ PFU. These uncertainty estimates were calculated only for the 25-curve validation subset and were not extended to all remaining curves in the database.

Digitisation uncertainty is nevertheless expected to have limited impact on derived competence metrics, because most studies reported mean log_10_ titres aggregated across individuals and the estimated extraction error was small relative to the log_10_ scale of variation in viraemia. When studies reported a mean viraemia curve directly in a table or figure, that mean curve was recorded as presented. When individual-level viraemia curves were available, we calculated the per-day mean log_10_ titre across individuals and retained only this mean curve in order to harmonise data representation across studies.

Measures of dispersion around the mean curves (e.g. standard errors, confidence intervals, or other error bars) were not retained as separate variables in the current version of the database because they were reported inconsistently across studies and could not be standardised across the full dataset.

Many studies also reported mortality curves, from which day-specific survival could be derived. Therefore, *sample_size_survival* should be interpreted as the number of individuals alive on the specific day represented by the row, rather than the final number surviving at the end of the experiment. However, because original studies did not always report the exact number of individuals contributing to each daily mean titre separately from the number surviving on that day, *sample_size_survival* should be regarded as the best available day-specific denominator and not necessarily the exact number used to calculate the reported mean titre.

The included studies primarily reported infection doses and viraemia titres using two types of units: plaque-forming units (PFU) and the tissue culture infectious dose infecting 50% of cultures (TCID50). Both PFU/ml and TCID50/ml quantify viral infectivity and virus titre, but they are based on distinct methodologies. To improve comparability between studies, we created a standardized variable in the database by converting TCID50-based values to PFU/ml using an approximate factor of 0.7, following the equation PFU/ml = TCID50/ml × 0.7^66–68^. Because the relationship between TCID50 and PFU varies with assay design and laboratory conditions, the applied conversion should be interpreted as a pragmatic standardization step that facilitates comparison across studies, while introducing additional uncertainty into the resulting PFU-equivalent values.

## Data Records

The datasets described in this study are archived in Zenodo as a persistent, versioned, citable release at https://doi.org/10.5281/zenodo.20024101^69^. The Zenodo repository contains two XLSX workbooks: **WNV_Host_Prevalence.xlsx** and **WNV_Host_Competence.xlsx**. Each workbook includes an observations table together with associated metadata, reference, and taxonomy sheets.

### WNV Host Prevalence Database

The file WNV_Host_Prevalence.xlsx contains four sheets: (i) observations, (ii) metadata, (iii) references, and (iv) taxonomy. The observations sheet contains row-level records extracted from published studies and publicly available surveillance reports. The metadata sheet defines all variables used in the observations table. The references sheet provides the complete bibliographic information for the cited sources, and the taxonomy sheet summarizes taxonomic harmonization and nomenclatural crosswalks (e.g., BirdLife, eBird, BirdTree).

In the prevalence database, diagnostic information is stored in long format, such that a single observation may correspond to more than one row when multiple assays were reported for the same species/sample record. The observations sheet includes the variables *diagnostic_method*, *assay_stage*, *workflow_design*, *diagnostic_group*, and *biological_interpretation*, which indicate the assay used (e.g., ELISA, molecular, VNT), its role within the study design (e.g., screening, confirmatory), the overall multi-assay design of the observation, its broader diagnostic category (e.g., serological, molecular), and the biological meaning of a positive result (e.g., past exposure, active infection). This structure allows users to distinguish records reflecting past exposure, recent exposure, or active infection, and to determine whether serological positives were supported by a confirmatory neutralization assay, which improves interpretation of ELISA-like results in the context of possible cross-reactivity. A summary of the relationship among these variables is provided in Table 1.

**Table 1:** Relationship between the standardised diagnostic fields used in the WNV host prevalence dataset. Categories shown correspond to the combinations currently observed in the dataset.

Diagnostic method	Assay stage	Workflow design	Diagnostic group	Biological interpretation
serological.ELISA	screening, independent	single_method, sequential, parallel	serology	past_exposure
serological.VNT	confirmatory, independent	single_method, sequential, parallel	serology	past_exposure
serological	independent	single_method, parallel	serology	past_exposure
HI	screening, independent	single_method, sequential, parallel	serology	past_exposure
IgC	independent	parallel	serology	past_exposure
MIA	independent	single_method	serology	past_exposure
IgM	independent	single_method, parallel	serology	recent_exposure
molecular_detection	independent	single_method, parallel	molecular	active_infection
IHC	independent	single_method	antigen	active_infection
virus_isolation	independent	single_method	isolation	active_infection

### WNV Host Competence Database

The file WNV_Host_Competence.xlsx contains four sheets: (i) observations, (ii) metadata, (iii) references, and (iv) taxonomy. The observations sheet contains row-level experimental infection data extracted from published studies. The metadata sheet defines all variables used in the observations table. The references sheet provides the complete bibliographic information for the cited sources, and the taxonomy sheet summarizes taxonomic harmonization across nomenclatural frameworks.

In the competence database, each row on the observations sheet corresponds to a specific day within an experimental infection curve. The variable *curve* identifies records belonging to the same infection time series, *day* indicates the day post inoculation, and *titer_pfu* stores the day-specific viraemia value. The variables *sample_size_original* and *sample_size_survival* indicate, respectively, the number of individuals inoculated at the start of the experiment and the number still alive on the row-specific day. The variable *PFU_dose* provides a standardized inoculation dose in PFU or PFU-equivalent units when this information could be recorded or converted from the source study. Additional fields record experimental context, including *inoculation_method*, WNV *lineage*, and *strain*, which are important for interpreting heterogeneity among infection experiments.

## Data Overview

### WNV Host Prevalence Database

**Fig. 1:**
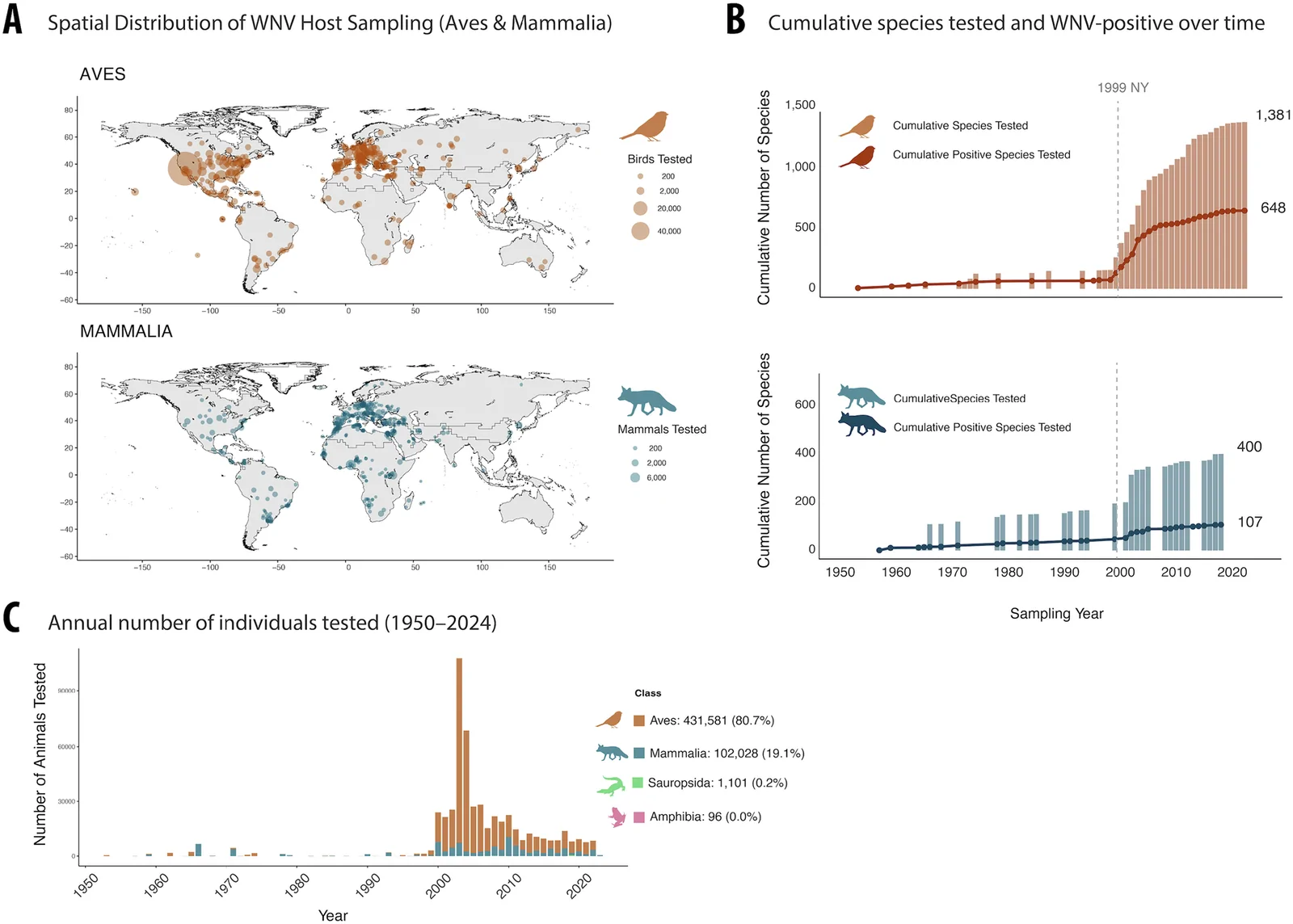
WNV Host Prevalence Database. **(A)** Global sampling locations of vertebrate hosts tested for West Nile virus (WNV) prevalence (1950–2024), shown separately for birds (Aves; top) and mammals (Mammalia; bottom). Circle size is proportional to the number of individuals tested at each site, combining molecular and serological assays. **(B)** Cumulative number of bird and mammal species tested (bars) and confirmed WNV-positive (lines) over time, displayed separately for birds (top) and mammals (bottom). **(C)** Annual number of individuals tested for WNV, stratified by vertebrate class.

**Fig. 2:**
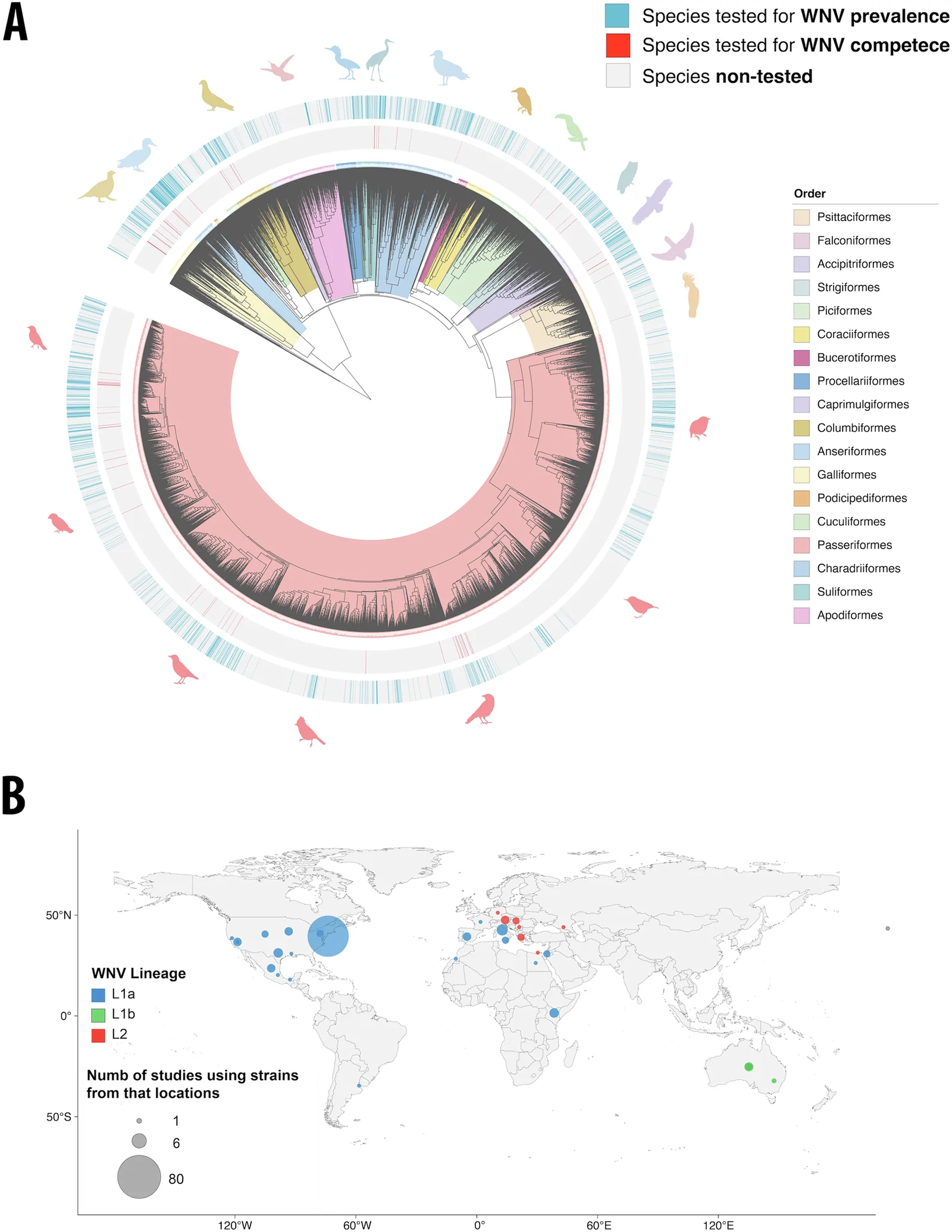
Phylogenetic coverage of WNV avian host evidence and geographic origin of WNV experimental strains. **(A)** Avian phylogeny from McTavish *et al*.^93^, comprising 9,239 species, annotated with study coverage from our two datasets. The outer annotation ring (blue) indicates bird species tested for WNV prevalence in field studies, whereas the inner annotation ring (red) indicates species evaluated for WNV competence in laboratory infection experiments. Grey denotes species not represented in either dataset. Coloured sectors highlight major avian orders. **(B)** Geographic distribution of WNV strains used in experimental infections (based on GenBank accession numbers when available). Point colour denotes WNV lineage, and point size is proportional to the number of studies using strains isolated from each location.

The WNV host prevalence dataset compiles records from 541 peer-reviewed studies and official reports from health agencies, covering 535,568 tested animals from 91 countries between 1950 and 2023 (Fig. 1). Birds represent the taxon most intensively surveyed, with 432,350 individuals tested (80.7%) in 1,377 species, followed by mammals with 102,198 individuals tested (19.1%) in 400 species. Within Aves, sampling is heavily concentrated in Passeriformes, which account for 679 tested species and 330,606 tested individuals (Table 2), followed by Charadiformes (97 species; 5,698 individuals), Anseriformes (87 species; 21,205 individuals) and Columbiformes (48 species; 21,080 individuals) (Fig. 2A). WNV testing was also recorded for 22 sauropsid, and 2 amphibian species. Among the 541 reviewed studies, 355 included avian hosts, yielding 10,093 records, nearly almost twice the 4,995 records compiled in the previous synthesis by Tolsá *et al*.^16^ obtained from 147 studies. The mammalian component includes 202 studies that contribute 1,501 records.

**Table 2:** Summary of bird orders included in the WNV host prevalence database, showing the number of species tested and the total number of individuals tested.

Order	Species tested	Species (pos.)	Species (neg.)	Individuals tested
Accipitriformes	78	41	37	8534
Anseriformes	87	39	48	21205
Apodiformes	22	7	15	970
Bucerotiformes	6	1	5	102
Caprimulgiformes	10	4	6	151
Casuariiformes	1	0	1	18
Charadriiformes	97	33	64	5698
Ciconiiformes	6	4	2	2349
Coliiformes	1	1	0	3
Columbiformes	48	29	19	21080
Coraciiformes	23	9	14	401
Cuculiformes	13	6	7	240
Falconiformes	24	15	9	3738
Galliformes	48	29	19	19117
Gaviiformes	3	0	3	6
Gruiformes	24	12	12	5717
Musophagiformes	2	1	1	24
Opisthocomiformes	1	0	1	4
Otidiformes	1	1	0	7
Passeriformes	679	313	366	330606
Pelecaniformes	37	27	10	3626
Phaethontiformes	2	0	2	180
Phoenicopteriformes	3	2	1	642
Piciformes	40	18	22	2127
Podicipediformes	5	5	0	88
Procellariiformes	7	0	7	253
Psittaciformes	46	13	33	439
Pteroclidiformes	1	0	1	1
Rheiformes	2	0	2	28
Sphenisciformes	2	1	1	94
Strigiformes	36	24	12	3834
Struthioniformes	1	1	0	12
Suliformes	16	10	6	1037
Tinamiformes	1	0	1	13
Trogoniformes	4	1	3	6

The spatial distribution of the host sampling is highly uneven across the globe. The surveillance effort has been concentrated in the Nearctic and Palearctic regions, while large tropical areas with high avian diversity, such as the Amazon basin, central Africa, and Southeast Asia, remain comparatively under-sampled (Fig. 4; Table 3). Consequently, apparent regional differences in host range and in the number of WNV-tested or WNV-positive taxa may partly reflect uneven surveillance effort rather than biological or ecological differences alone. This pattern is also temporally structured: sampling increased markedly after the emergence of WNV in New York in 1999 and its subsequent spread across the Americas, which stimulated major surveillance and research efforts. By contrast, earlier studies were relatively sparse and concentrated mainly in Africa, the Middle East, and eastern Europe, where WNV had already been historically recognized.

**Table 3:** Sampling coverage of terrestrial bird species by biogeographic realm according to §ekercioğlu *et al*.^92^. Species occurring in multiple realms contribute to the total of each corresponding realm; therefore, totals across realms are not additive.

Biogeographic realm	Total bird species	Sampled bird species	Sampled (%)	Unsampled species
Nearctic	732	640	87.4	92
Palearctic	1041	687	66.0	354
Afrotropical	1985	257	12.9	1728
Indomalayan	2021	76	3.8	1945
Neotropical	4303	493	11.5	3810
Australasian	1382	37	2.7	1345
Madagascan	264	34	12.9	230

**Fig. 3:**
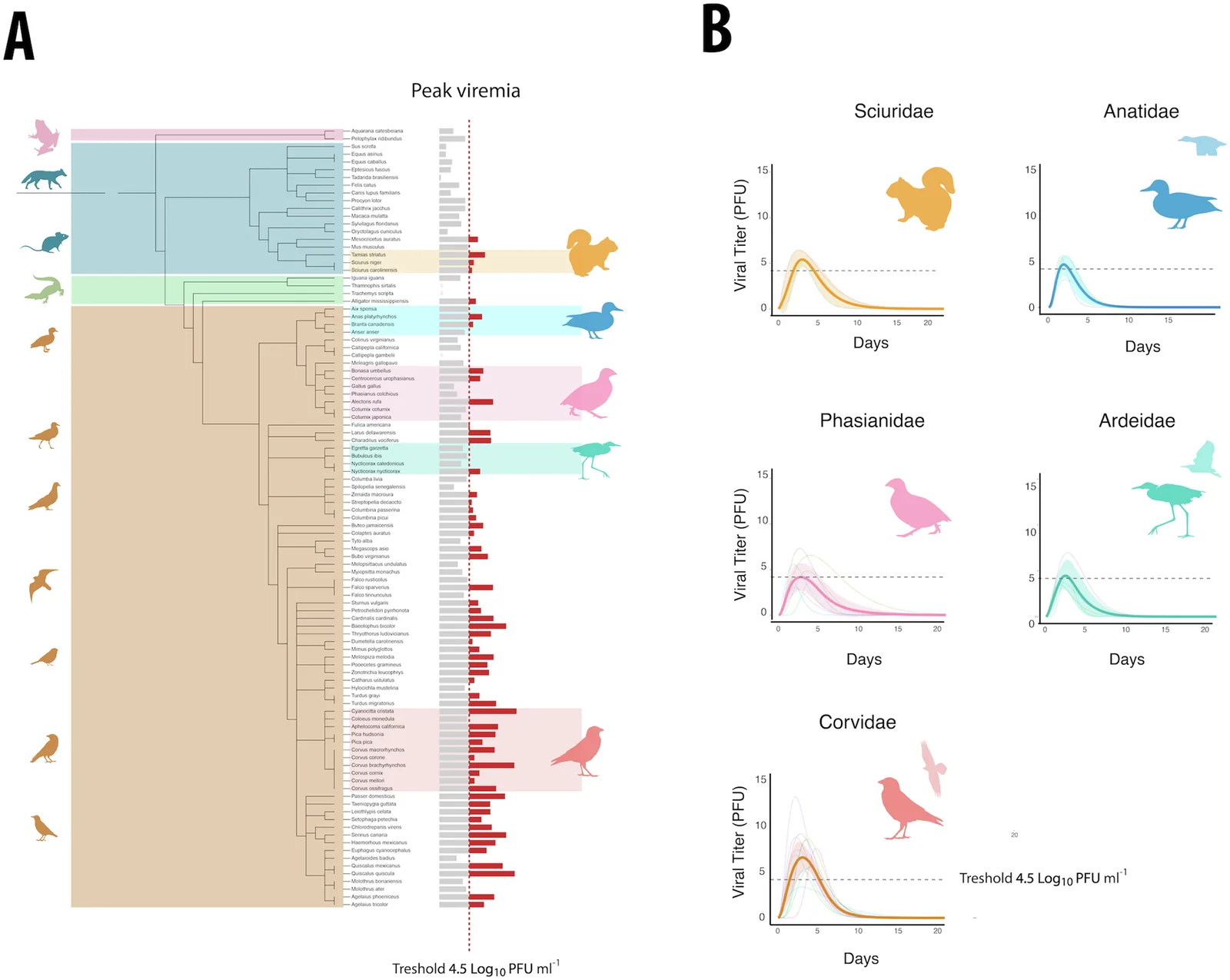
Peak viraemia and reconstructed viraemic curves from experimental infections. **(A)** Peak viraemia (log_10_ PFU mL^−1^) reported for experimentally infected host species, shown across major vertebrate groups. The dashed line indicates the commonly used infectivity threshold of 10^4.5^ PFU mL^−1^ in blood, above which feeding mosquitoes are more likely to become infected during a blood meal. **(B)** Examples of reconstructed viraemic curves (viral titre, PFU mL^−1^) for representative host families, illustrating the time-resolved experimental data curated in the WNV host competence database.

**Fig. 4:**
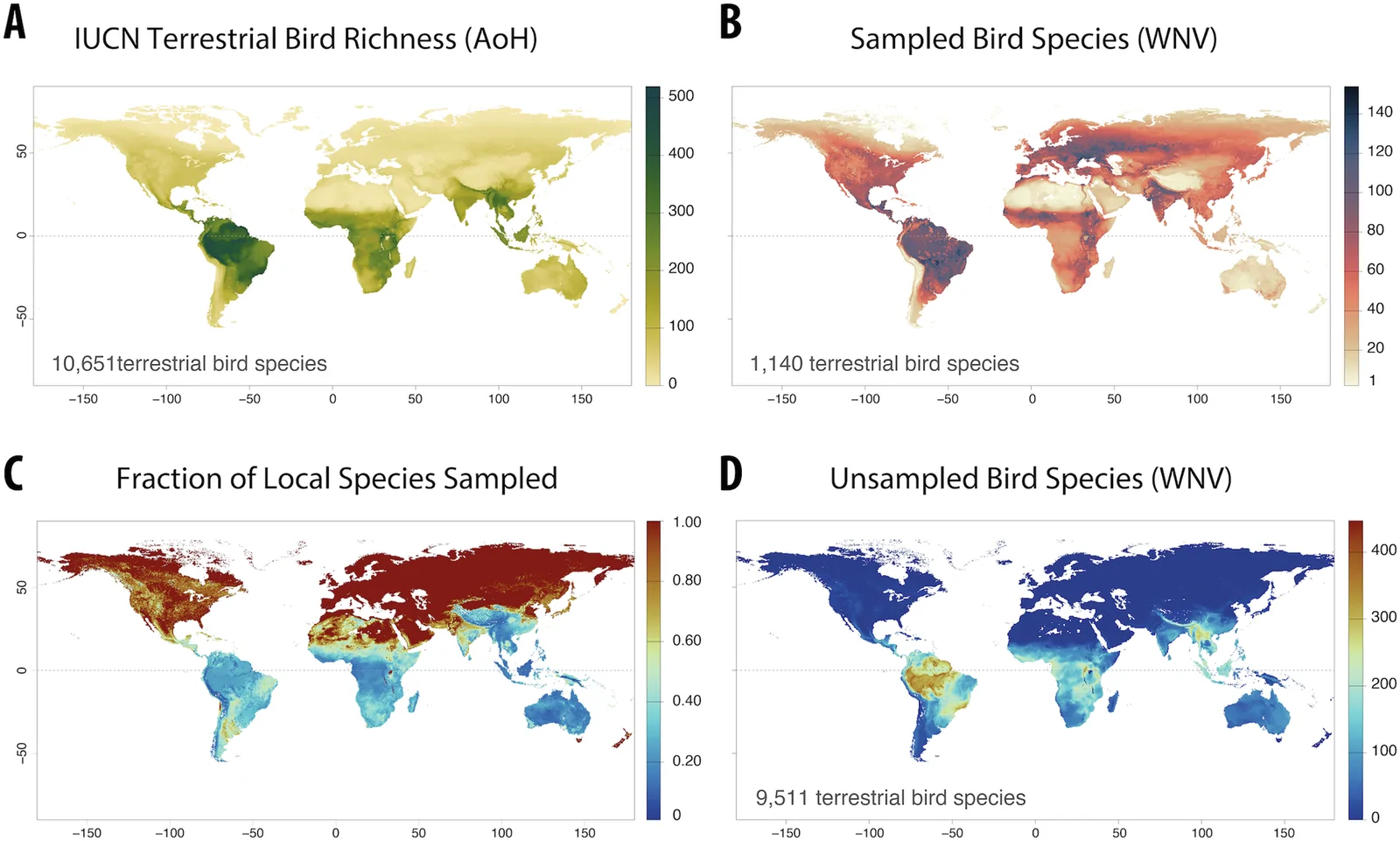
WNV host prevalence database: global coverage and gaps in avian sampling. **(A)** Terrestrial bird species richness derived from IUCN Areas of Habitat (AoH) maps^75^ (https://doi.org/10.5061/dryad.02v6wwq48). **(B)** Spatial distribution of bird species included in the WNV host prevalence database (tested species, irrespective of WNV outcome). **(C)** Fraction of locally occurring bird species sampled for WNV, representing the proportion of local avian diversity surveyed in each area (panel (B) / panel (A)). **(D)** Estimated number of locally occurring bird species not yet sampled for WNV (unsampled richness), calculated as panel (A) minus panel (B).

Our systematic review identified a total of 647 bird species that have tested positive for WNV, representing 47% of the avian species screened. These detections should not be interpreted uniformly in epidemiological terms. Depending on the diagnostic method used, positive records may indicate past exposure, recent exposure, or active infection, but do not demonstrate transmission competence or reservoir status.

Of these, 284 species (43.8%) were confirmed positive by molecular assays (e.g. RT-PCR), which provide stronger evidence of active infection and potential viral shedding. They therefore may indicate a higher likelihood of reservoir competence^16^. However, such inferences remain provisional and should be validated under controlled experimental conditions. Molecular detection alone does not demonstrate that a species can develop sufficient viremia to infect mosquitoes and contribute to future transmission.

By contrast, positivity detected exclusively by serological methods (e.g., IgM/IgG ELISA or VNT) reflects primarily prior exposure and does not necessarily imply the capacity to transmit the virus to mosquitoes^16^. Instead, evidence for transmission competence must be derived from the WNV Host Competence database generated from controlled experimental infection studies. Refer to the “Usage Notes” section for more details on the number of species tested using different validation methods (serological or molecular) and how these should be considered when using the database. Furthermore, 111 mammalian species (27.8%) were reported as WNV-positive. Most mammalian detections were based on serological evidence (ELISA and/or virus neutralisation tests), with comparatively fewer reports supported by molecular confirmation, reflecting past exposure rather than active infection or transmission potential. The majority of the mammalian positives reported occurred after the introduction of WNV and its subsequent spread throughout the American continent (Fig. 1).

### WNV Host Competence Database

The systematic review resulted in the inclusion of data from 113 peer-reviewed studies that reported a total of 362 viraemia curves, representing 103 different species. Birds were the most represented group, with 80 species spanning 12 orders and 30 families, and a substantial sample size of 1,925 experimentally tested individuals. Within Aves, Passeriformes, Galliformes, and Columbiformes were the orders more studied, with 39, 12 and 6 species, respectively. Mammalia includes 16 species from 12 families in 7 orders, with 237 tested animals. Reptilia and Amphibia are represented by fewer species and smaller sample sizes, with Reptilia comprising 4 species and 97 individuals, and Amphibia contributing 2 species and 74 tested animals.

The raw experimental data allow identification of species whose viraemia peaks exceed the commonly used infectivity threshold of 10^4.5^–10^5^ PFU in the blood, above which feeding mosquitoes are more likely to become infected (Fig. 3A)^70^. This threshold should be interpreted as an approximate reference value rather than a universal cutoff, as it has been derived mainly from studies involving *Culex pipiens*. The relationship between host viraemia and mosquito infection probability can vary between vector species, between populations within a species, and with environmental conditions such as temperature, which affects viral replication in mosquitoes. Moreover, by extracting time-resolved titre measurements in a standardised format, our database supports reconstruction and/or modelling of species-specific viraemia trajectories and associated survival profiles (Fig. 3B). This facilitates the derivation of quantitative competence metrics, including peak titre and timing, duration of viraemia above a chosen infectivity threshold, and cumulative infectiousness (e.g., area under the curve above threshold), and provides inputs for subsequent transmission modelling analyses.

The experimental conditions represented in the competence database are heterogeneous with respect to inoculation route, inoculated dose, and viral genotype. Across the 362 infection curves, needle inoculation was the most common exposure route, followed by mosquito-mediated exposure and oral exposure. Comparable PFU-dose information was available for all needle-inoculation curves, but was missing for part of the mosquito-mediated and oral-exposure experiments, reflecting incomplete reporting of comparable delivered-dose information in these study designs. In mosquito-mediated experiments, this usually occurred because the virus dose offered to mosquitoes was not reported, whereas in oral-exposure experiments it often reflected the absence of information on the infectious dose contained in the prey item or food material. Moreover, when the PFU-dose was reported for these routes, it represents the dose offered to mosquitoes or contained in prey, not necessarily the dose effectively transmitted to or ingested by the vertebrate host. Mosquito-mediated exposure experiments involved at least 10 reported mosquito taxa from two genera (*Aedes* and *Culex*), further emphasizing heterogeneity in vector-mediated protocols across regions. A summary of these experimental design conditions, including the number of studies, curves, host species by vertebrate class, and PFU-dose ranges by inoculation route, is provided in Table 4.

**Table 4:** Summary of experimental design conditions represented in the WNV Host competence database by inoculation route. PFU-dose statistics were calculated only for curves with available comparable dose information.

Route	Studies	Curves	Bird	Mammal	Other	PFU avail.	Min	Median	Max
Needle inoculation	107	304	66	14	6	304	2.00	1.58 × 10^3^	3.00 × 10^8^
Mosquito-mediated exposure	15	50	31	8	2	38	170	1.00 × 10^5^	1.68 × 10^9^
Oral exposure	5	8	2	3	1	7	100	2.00 × 10^3^	6.31 × 10^3^

Most species investigated in controlled laboratory infection experiments were also represented in field-based prevalence sampling (Fig. 2A). Only two experimentally tested species lacked the corresponding prevalence records in the wild: *Corvus mellori*(little raven, Australia) and *Chlorodrepanis virens* (Hawai’i ’amakihi, Hawai). Inclusion of GenBank accession numbers, together with standardised metadata on viral lineage, isolation year, and isolate location, enables detailed characterisation of strains used in experimental infections. The large majority of experimental studies have used WNV lineage 1 strains (96.5% of studies), predominantly isolates derived from the NY99 strain, whereas lineage 2 strains have been used comparatively less frequently (8.0% of studies), and lineage 1b and Kunjin-related isolates have been rarely included (2.7% and 0.9% of studies, respectively) (Fig. 2B). These annotations provide a transparent basis for assessing the comparability of experimental outcomes across studies, evaluating the representativeness of commonly used strains relative to circulating viruses in different regions, and guiding the design of future experiments aimed at testing lineage- and strain-specific differences in host infectiousness.

## Technical Validation

All host records included in the database come from peer-reviewed scientific articles, books, and official reports, ensuring rigorous data validation. Additional data from publicly accessible databases managed by health agencies were incorporated if they provided verifiable, active links, and sufficient methodological details according to established inclusion criteria. In particular, certain records previously reported by Tolsà-García *et al*.^16^ of health agencies in the United States and Mexico were excluded, as the original data links were no longer available and requests to access these data were unanswered.

To maintain consistency and ensure robust data quality, we strictly adhered to the standardization and harmonization criteria outlined in the materials and methods. In particular, studies were excluded if they only reported positive cases without providing complete details on the species and the numbers of negative individuals tested. Each entry in the data set is accompanied by its bibliographic source, enabling users to assess the validity of the data and facilitating future data reuse. All references cited are fully documented, providing direct access to the original studies.

Given the dynamic nature of bird taxonomy, avian host classifications follow an integrative approach aligned with the AVONET dataset^50^, incorporating multiple taxonomic treatments to facilitate compatibility with ecological trait databases, phylogenetic analyses, GBIF distribution observations, and eBird abundance maps. Specifically, we applied the taxonomic crosswalk framework of Tobias *et al*.^50^, integrating BirdLife International^46^, eBird^48^, and BirdTree^47^, with additional verification and updates performed via Avibase^71^. Taxonomic harmonization followed a hierarchical decision process. First, when a BirdLife/Avibase taxon had a unique one-to-one match in BirdTree or eBird, that corresponding name was retained directly. Second, when multiple candidate matches existed, we prioritized the option that preserved the BirdLife/Avibase name exactly whenever that same-name alternative was available, in order to maximize consistency with the primary avian taxonomic backbone used in the database. Third, when no exact same-name option existed in the target framework, we retained the closest taxonomic equivalent available for interoperability, while preserving all alternative candidate matches explicitly in the taxonomy table. To maintain transparency, the ‘Taxonomy` sheet stores both the selected harmonized name and the full set of alternative candidate names, together with match-type fields indicating whether the correspondence was one-to-one or one-to-many. This allows users to identify ambiguous cases and, if needed, apply alternative taxonomic decisions in downstream analyses. Mammalian hosts were standardized using the Mammal Diversity Database^53,72^ and the GBIF Backbone Taxonomy^49^, thus ensuring precision by retrieving accepted scientific names along with their corresponding taxon IDs from the GBIF.

Given that pathogen host ranges are inherently dynamic, influenced by pathogen evolution, diversity, and spread, together with intensity of surveillance, and considering the increased research attention to WNV following recent outbreaks in Europe^2^, our goal is to regularly maintain and expand the database. This will be achieved through systematic reviews of the literature and direct contributions from the research community. We encourage researchers to report any updates or corrections to the corresponding author (alex.richter@ceab.csic.es) to ensure accuracy and ongoing data improvement.

## Usage Notes

### Linking to External Datasets and Identifying Sampling Gaps

To facilitate integration with other biodiversity and ecological datasets, the compiled dataset includes multiple standardised species identifiers (e.g., BirdLife, GBIF, eBird, BirdTree). This enables cross-referencing across nomenclatural systems and supports linking with widely used resources such as AVONET^50^, EltonTraits^73^, BirdLife species distribution maps^74^, IUCN Areas of Habitat maps^75^, and eBird abundance estimates^76^. Combined analyses of host testing records for WNV with species distributions, habitat associations, and abundance estimates can support spatially explicit assessments of host ecology, biogeography, and community-level drivers of virus circulation and spillover risk. As an example, overlaying host testing records with regional species assemblages allows quantification of the proportion of local avian diversity that has been sampled, thus identifying geographic gaps in surveillance and prioritising regions for targeted field sampling (Fig. 4). Large areas in the Southern Hemisphere and Southeast Asia remain poorly represented, including the Amazon basin, the Congo rainforest, and parts of Indonesia and Papua New Guinea, where sampling coverage is low relative to local avian richness. This uneven effort, concentrated in the Palearctic and Nearctic realms, may bias current inference on host susceptibility, host range, and transmission risk. In particular, apparent host-range expansion in better-sampled regions may partly reflect surveillance intensity rather than true ecological generalism, highlighting the need for more geographically balanced surveillance and for caution when comparing host-range patterns among regions.

### Interpretation of Diagnostic Outcomes

The WNV host prevalence database includes studies reporting WNV detection using a range of diagnostic techniques, primarily molecular methods and serological assays. When interpreting these results, particularly in comparative or meta-analytical contexts, it is important to recognise that detection outcomes depend on the diagnostic method applied. Each method targets a distinct stage of the infection process, ranging from early-phase viraemia (detected via RT-PCR) to later-stage immune responses (detected via IgM or IgG ELISA or VNT). Importantly, diagnostic detection windows differ markedly: RT-PCR has a short detection window, typically restricted to the acute viraemic phase over a few days, whereas antibodies can remain detectable for many months to more than a year^77,78^. Because serological, molecular, antigen-based, and isolation-based records capture different stages of the infection process and operate in different temporal windows, raw prevalence values should not be directly compared across diagnostic categories without appropriate stratification. To reduce misinterpretation, users can filter records by *diagnostic_method*, *diagnostic_group*, *assay_stage*, *workflow_design*, or *biological_interpretation*, depending on whether the analytical objective is to distinguish assay type, biological interpretation, or diagnostic workflow.

Among the studies compiled here, most relied on a single diagnostic approach (*n* = 400/541,  ~73.9%), most commonly serology alone—particularly virus neutralization tests (VNT; *n* = 145, 26.8%) and ELISA (*n* = 139, 25.6%)—or molecular detection alone (e.g., RT-PCR; *n* = 115, 21.2%). A fraction combined two diagnostic methods (*n* = 127,  ~23.5%). Combinations that integrated molecular and serological evidence were rarer (molecular detection + ELISA: *n* = 19, 3.51%; molecular detection + VNT: *n* = 10, 1.84%). Combining molecular and serological tools is particularly valuable for interpretation, as it helps distinguish active infection from past exposure, thus improving temporal inference of transmission dynamics and reducing ambiguity when prevalence estimates span different phases of infection.

Diagnostic specificity is an important consideration when interpreting WNV surveillance data in the WNV host prevalence database. Molecular assays (RT-PCR and RT-qPCR) are highly specific and are commonly used as reference methods for detecting acute infection^79,80^. Serological assays require more cautious interpretation: ELISA-based methods are sensitive but may show cross-reactivity with antibodies against closely related flaviviruses, depending in part on the antigen used^81^. Cross-reactivity is particularly relevant among members of the same serocomplex (e.g., WNV, Usutu virus (USUV), and Japanese encephalitis virus (JEV))^82^. Virus neutralisation tests (e.g., PRNT) provide greater specificity and are often used for serological confirmation, although some cross-neutralisation can still occur^79,80^. Where co-circulating flaviviruses are expected, parallel testing against WNV and related viruses can improve attribution of seropositivity, avoiding under- or overestimation of WNV circulation and host range within a given region. In addition, IgM is generally considered less cross-reactive than IgG and is often used to support inference on recent infection, as IgM typically rises and wanes more rapidly than IgG.

**Fig. 5:**
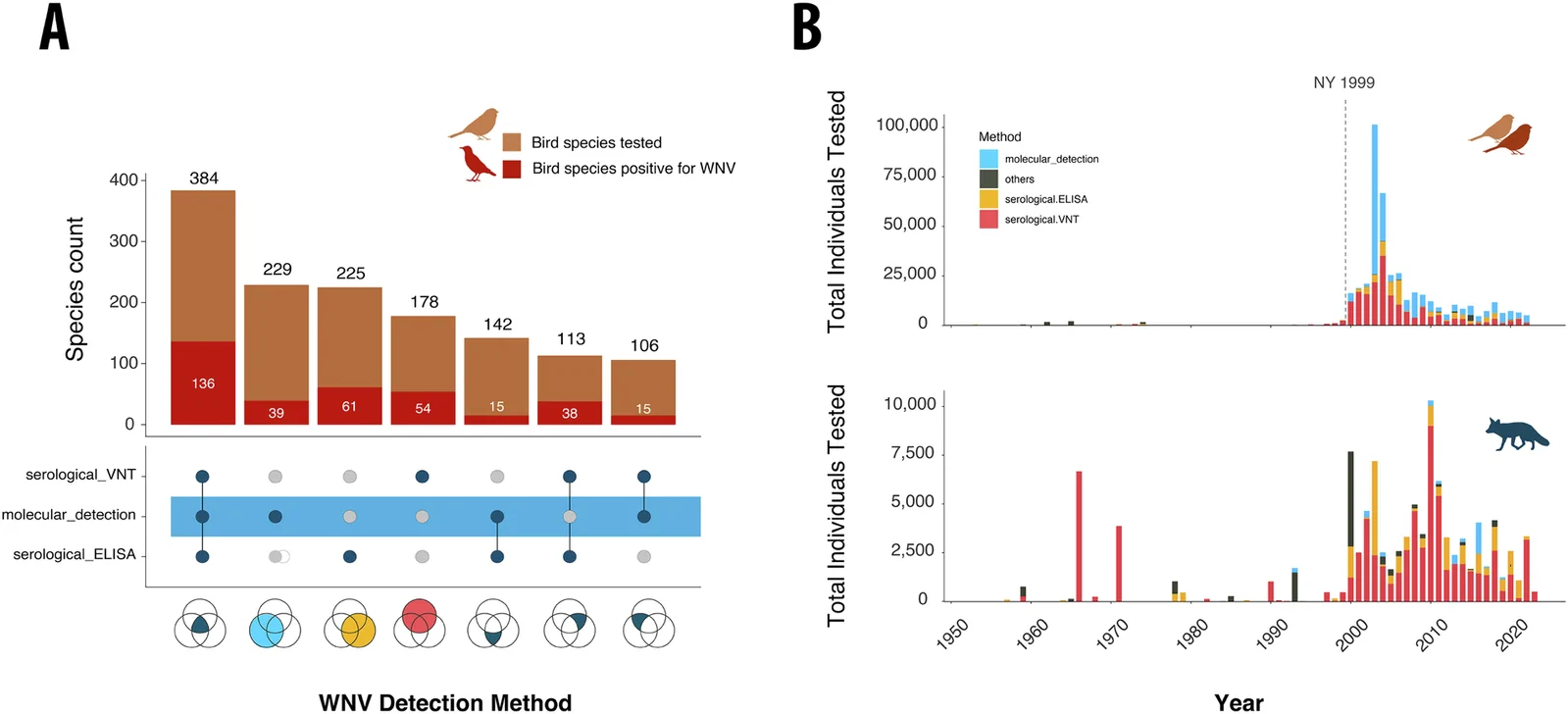
WNV host prevalence database: diagnostic methods across taxa and time. **(A)** Number of bird species screened for WNV and number of birds reported WNV-positive, stratified by the three most commonly used diagnostic approaches (molecular detection, serological ELISA, and virus neutralisation tests [VNT]) and their combinations. The matrix and intersection diagram summarise overlap among methods, highlighting species tested using single versus multiple diagnostic approaches. **(B)** Annual number of individuals tested for WNV in birds (top) and mammals (bottom) from 1950–2024, with stacked bars indicating the contribution of each diagnostic category. For individuals tested using multiple methods within a study, counts were assigned to a single category using a hierarchical rule favouring confirmatory/specific assays (molecular detection  > VNT > ELISA), to avoid double counting. Molecular assays primarily capture acute infection, whereas serological approaches reflect prior exposure; the “other” category includes less frequently used assays (e.g., immunohistochemistry) The dashed line marks 1999, corresponding to the first detection of WNV in New York, USA.

In birds, serological neutralization assays—considered the gold standard—along with molecular techniques^79,80^, are the most commonly used. The use of the different methods has changed over time, with serological neutralization assays dominating the early 2000s and molecular testing increasing thereafter, now accounting for a large share of avian testing in recent years (Fig. 5B). Mammals show a different pattern: neutralization assays remain predominant across decades, with molecular methods used more sparingly and without the sustained rise seen in birds.

The use of the diagnostic method is therefore highly uneven between species. Only 27.8% (384/1,377) of avian species have ever been assessed with all three diagnostic approaches, while substantial fractions were tested exclusively by neutralization assays (21.1%) or exclusively by molecular methods (16.6%) (Fig. 5A). This imbalance has two implications. First, cross-species comparisons of “positivity” are not directly comparable unless the choice of method is taken into account because, as explained before, molecular methods primarily capture current or very recent infection (and potential shedding), while serology reflects past exposure with a much longer detection window. Second, the distribution of methods across species is unlikely to be random: the higher share of positives observed in molecular-only subsets plausibly reflects targeted sampling of suspected reservoirs or outbreak contexts rather than intrinsic differences in test performance (i.e., ascertainment bias). Methodological differences must be taken into account when inferring host susceptibility and prevalence. For analyzes using this dataset, we recommend: (i) stratifying or weighting by diagnostic method; (ii) conducting sensitivity analyzes contrasting molecular-confirmed versus serology-only records; and (iii) reporting method-specific prevalence to avoid conflating past exposure with current infection.

Accordingly, the prevalence database should be interpreted as documenting the observed field host range of WNV susceptibility (exposure and infection), rather than the functional transmission host range of the virus. Reservoir or amplification status cannot be inferred from prevalence data alone and requires complementary experimental evidence on viraemia and infectiousness to mosquitoes.

### Data Considerations for Host Competence Modelling

Experimental infection data provide the basis to estimate and characterise host-specific viraemia curves and, consequently, inferring potential host contributions to WNV amplification and transmission. However, comparisons of viraemia dynamics and competence metrics between species and studies should explicitly account for differences in experimental design, as these can strongly influence estimated outcomes. Key variables include the inoculated dose (typically reported in PFU/mL or equivalent units), the route of inoculation (e.g., syringe inoculation, mosquito feeding, or oral exposure through infected prey), and viral factors such as lineage and strain^24^.

Importantly, the reported inoculated doses are not always directly comparable between the inoculation methods. Under needle-based inoculation (syringe), the dose administered can typically be approximated as the product of inoculum concentration and injected volume, and therefore closely reflects the amount of virus delivered to the host. In contrast, when infection is achieved through mosquito feeding or oral exposure, the reported titre usually corresponds to the virus dose offered to mosquitoes or the infectious titre of prey material, rather than the quantity effectively transmitted to the vertebrate host. Experimental and entomological studies have also shown that inoculum administered during mosquito feeding varies between vector species and that mosquito saliva can modulate infection dynamics, including enhanced viraemia and tissue titres in vertebrate hosts^83,84^. Therefore, this distinction should be considered in downstream analyses that aim to relate inoculated dose, inoculation route, and viral genotype to resulting viraemia curves and inferred transmission potential.

The viral genetic background is a critical source of heterogeneity in experimental results^85^. Experimental infections in the house sparrow (*Passer domesticus*) have shown that transmission phenotypic traits, including the magnitude of viraemia, tissue dissemination, and viral shedding, can differ substantially among WNV strains^24,86,87^. In particular, such differences occur not only between lineages but also among strains within the same lineage. For example, NY99 (lineage 1a) has been associated with higher and more persistent viraemia than other strains of the same lineage, or of lineage 2 strains^87^. Although multiple WNV lineages have been described globally^88,89^, experimental infections have focused predominantly on lineages 1a and 2, which may limit inference in regions where distinct strains circulate or where multiple lineages co-circulate.

To facilitate robust comparative analyses, the database records, when available, the inoculated dose and units, the inoculation method, the lineage and strain identity (including GenBank accession numbers), and isolate year and location. These annotations enable sensitivity analyses and model-based reconstructions that explicitly account for experimental conditions, improving comparability between studies and supporting the recalculation of viraemia curves and competence metrics under standardised assumptions^24,90,91^. Because inoculation route, inoculated dose, lineage, and strain can all influence observed viraemia dynamics and survival, users should account for these experimental design variables when deriving host competence metrics or comparing species across studies. In particular, curves generated under markedly different exposure conditions should not be pooled uncritically. Depending on the analytical objective, these sources of heterogeneity may be addressed through stratified analyses or through statistical or modelling approaches that separate experimental effects from intrinsic biological differences among host species. This is especially relevant when estimating metrics such as peak viraemia, duration of infectiousness, or area under the viraemia curve, which may be systematically affected by experimental protocol.

For users interested in reconstructing experimental time series, each row in the competence database represents a specific day within an infection curve, identified by a unique *curve* identifier. Viraemia curves can therefore be reconstructed by grouping rows by *curve* and ordering them by *day*, using *titer_pfu* as the day-specific viraemia value. These reconstructed trajectories may then be fitted using different mathematical functions to characterise viraemia dynamics, for example with Wood’s model, logistic or Gompertz-type curves, or more flexible spline-based approaches, depending on the structure of the data and the intended downstream analysis. Survival trajectories can be reconstructed in the same way using *sample_size_survival* across days. The variable *sample_size_original* indicates the initial number of inoculated individuals in that experimental curve. Users should note, however, that *sample_size_survival* reflects the number of individuals alive on a given day and not necessarily the exact number contributing to the reported mean titre at that time point, as some studies sampled only a subset of surviving individuals on each day.

## Data Availability

The datasets can be downloaded from Zenodo under the CC 4.0 license at https://doi.org/10.5281/zenodo.20024101.

## Code availability

The code for the viremic and mortality curve reconstruction examples are publicly available through the GitHub repository (alexRichterBoix/WNV_host_competence_-_host_range).
